# Cross-sectional and longitudinal associations between Life’s Essential 8 and frailty in community-dwelling older adults

**DOI:** 10.3389/fpubh.2025.1730769

**Published:** 2026-01-05

**Authors:** Zhiqiang Ren, Gang Liu, Jie Zhao, Qishan Ma, Xuan Zou, Xiaoheng Li, Xudong Liu, Wenjing Zhao

**Affiliations:** 1 School of Public Health and Emergency Management, School of Medicine, Southern University of Science and Technology, Shenzhen, China; 2 SUSTech Homeostatic Medicine Institute, School of Medicine, Southern University of Science and Technology, Shenzhen, China; 3 Shenzhen Center for Disease Control and Prevention, Shenzhen, China; 4 Shenzhen Maternity & Child Healthcare Hospital, Shenzhen, China; 5 Department of Epidemiology and Health Statistics, School of Public Health, Guangdong Pharmaceutical University, Guangzhou, China

**Keywords:** Life’s Essential 8, frailty, older adults, community-dwelling, real-world data

## Abstract

**Objective:**

The American Heart Association recently updated an algorithm—Life’s Essential 8 (LE8)—for evaluating cardiovascular health (CVH). However, the cross-sectional and longitudinal associations between LE8 score and frailty among community-dwelling older adults remain unclear.

**Methods:**

We analyzed the health examination database of community-dwelling older adults aged 65 and above in an administrative district of Shenzhen, China, from 2018 to 2022, based on China’s National Essential Public Health Service Package (NEPHSP). The LE8 score was calculated as the average of eight metrics: nicotine exposure, physical activity, diet, sleep health, body mass index, blood lipids, blood glucose, and blood pressure. A 30-item frailty index (FI) was constructed that excluded deficits related to cardiovascular disease. The data were analyzed using logistic regression models and Cox proportional hazards models.

**Results:**

A total of 59,654 older adults participated in the cross-sectional analyses, with 57,742 providing longitudinal data. Over a mean follow-up period of 1.21 years, 1,365 incidents of frailty were identified. In the cross-sectional analysis, a higher LE8 score was associated with lower odds of frailty. In the longitudinal analysis, a higher LE8 score was associated with reduced hazard ratios for frailty incidence. These associations remained significant after adjusting for covariates such as age, sex, registration, marital status, educational level, drinking habit, hypertension, type 2 diabetes, heart disease, stroke, and total cholesterol.

**Conclusion:**

Better CVH was significantly associated with a lower frailty risk among community-dwelling older adults. Our findings highlight the importance of enhancing CVH as a preventive measure against frailty within the primary healthcare system.

## Introduction

Frailty, an age-related syndrome marked by a decline in homeostatic reserve and heightened vulnerability to stressors, is prevalent among older people (1). It was estimated by a meta-analysis that the incidence of frailty was 43.4 new cases per 1,000 person-years (2). As the aging population rapidly expands, the prevalence of frailty among older adults worldwide is increasing dramatically (3). Frailty increases the risk of various adverse health outcomes, including impaired mobility, falls, hospitalizations, and mortality (4). Consequently, elucidating the underlying causes of frailty is essential for developing effective preventive strategies to reduce its prevalence and alleviate the burden on primary healthcare.

In 2022, the American Heart Association (AHA) updated the operational tool for cardiovascular health (CVH) assessment, known as Life’s Essential 8 (LE8), to address existing cardiovascular disease (CVD) and related risk factors, thereby promoting the health of both populations and individuals (5). The LE8 score has been well validated as a composite and quantified measurement of CVH (6) and has demonstrated inverse associations with adverse outcomes, such as diabetes, sarcopenia, dementia, and all-cause and CVD mortality (7–10). Chronic conditions, such as CVD (including atrial fibrillation, heart failure, and coronary heart disease), are more likely to contribute to the incidence of frailty (11). Several of the LE8 metrics, including physical inactivity, unhealthy dietary habits, insufficient sleep, lipid abnormalities, and glucose metabolism disorders, are known to contribute to a state of chronic low-grade inflammation and metabolic dysregulation (12–15). These processes promote the release of pro-inflammatory factors, which are implicated in both cardiovascular risk and the pathogenesis of frailty. By reducing physiological reserve capacity and promoting endothelial dysfunction and atherosclerosis, these mechanisms impair the body’s ability to cope with stressors, thereby contributing to the onset of frailty (16). Therefore, managing and improving CVH through the LE8 metrics is hypothesized to mitigate these inflammatory and metabolic pathways, offering a preventive strategy against frailty. One previous study examined the cross-sectional association between LE8 and frailty among American cancer survivors (17). However, the evidence on the association between LE8 and frailty in the community-dwelling older adults is still lacking, and it remains unclear whether the LE8 score impacts the incidence of frailty.

We hypothesized that a higher LE8 score, reflecting better CVH, would be significantly associated with lower odds of frailty at baseline and reduce the risk of frailty incidence during follow-up. In this study, we aim to investigate both the cross-sectional and longitudinal associations between the LE8 score and frailty among community-dwelling older adults. Understanding these associations helps provide population-based evidence supporting the role of CVH maintenance in preventing frailty in older adults.

## Materials and methods

### Study setting and participants

The data in this study were extracted from the health examination database routinely collected in an administrative district of Shenzhen, China, from 2018 to 2022, based on China’s National Essential Public Health Service Package (NEPHSP). The study protocol has been described in detail elsewhere (18). Briefly, the NEPHSP is a fundamental public health program aimed at providing basic health services to all Chinese residents at the primary healthcare level. Older adults aged 65 and above enrolled in the NEPHSP were annually subjected to interviews, physical examinations, and blood tests administered by highly trained medical personnel at their local community healthcare centers. The health examination database included sociodemographic and lifestyle characteristics, health-related questions, physiological measurements, and laboratory tests. This study was approved by the Ethics Committee of Southern University of Science and Technology. Since the study used anonymized real-world data, approval was obtained for a waiver of individual consent.

Among the 60,991 participants at baseline, we initially excluded those who self-reported dementia or mental diseases (*n* = 89), as well as participants with missing data on the LE8 score (*n* = 1,090) and those with missing data on frailty index (*n* = 158). This resulted in 59,654 participants for our cross-sectional analyses. Next, we excluded 1,880 older adults with frailty at baseline and 32 participants with missing follow-up data on the outcome, leaving 57,742 participants for the longitudinal analysis (Supplementary Figure S1).

### Measurement of LE8

According to the AHA’s LE8 algorithm with previous studies in China, we created an LE8 score using four health behaviors (nicotine exposure, physical activity, diet, and sleep health) and four health factors (body mass index [BMI], blood lipids, blood glucose, and blood pressure) (5, 19). Minor adaptations were made to the scoring categories of health behaviors to reflect data availability in the NEPHSP database, using AHA definitions (5) and the Kailuan study (19), which reported a modified LE8 score for the Chinese population. The information on health behaviors was collected using standardized self-reported questionnaires. Weight and height assessments were obtained through physiological measurements, from which BMI was calculated by dividing weight (kg) by the square of height (m). The fasting blood glucose, total cholesterol, low-density lipoprotein cholesterol, and high-density lipoprotein concentrations were measured at the medical laboratory of community healthcare centers. Blood pressure was measured by trained staff using a validated digital portable monitor. Each of the eight metrics above was scored from 0 to 100, and we averaged the component score to derive the overall LE8 score, ranging from 0 (worst health) to 100 (best health). The detailed methods and criteria are presented in Supplementary Table S1.

### Measurement of frailty

Frailty was defined using a 30-item frailty index (FI) created according to standard procedures outlined by Theou (Supplementary Table S2) (20). An FI requires a sufficient number and diversity of deficits, including diseases, symptoms, functional impairments, and laboratory abnormalities. We selected 30 health variables that covered multiple physiological domains to meet the established criteria. The FI is calculated as the proportion of deficits in an individual relative to the total number of deficits at a certain time point. The FI ranges from 0 to 1, with a higher FI indicating a greater level of frailty. We excluded deficit items related to CVD, including conditions such as stroke, heart attack, and hypertension; symptoms such as headache; items related to type 2 diabetes mellitus, including symptoms such as fatigue and weakness and indicators such as hemoglobin A1C; and total cholesterol levels. With reference to previous studies, participants were categorized into two groups: non-frailty (0 ≤ FI < 0.20) and frailty (FI ≥ 0.20) (21).

### Covariates

The covariates included age (continuous), sex (female or male), residency status (local or non-local), educational level (less than high school, high school or equivalent, or college and above), marital status (married or other), drinking habits (never, seldom, or daily), total cholesterol (continuous), and history of hypertension (yes or no), type 2 diabetes (yes or no), heart disease (yes or no), and stroke (yes or no). These covariates were assessed through standardized self-reported questionnaires and laboratory blood tests.

### Statistical analysis

Baseline characteristics were presented as means with standard deviation (SD) for continuous variables and frequencies with proportions (%) for the categorical variables. We expressed the LE8 score in sex-specific quartiles because women generally report healthier diets and have lower nicotine exposure than men (22, 23). To access differences in baseline characteristics among participants according to sex-specific quartiles of the LE8 score, we used the Kruskal–Wallis rank-sum test or Pearson’s *χ^2^* test, as appropriate. Missing data for covariates were imputed using the modal value for categorical variables and the median for continuous variables.

We investigated the cross-sectional associations between the quartiles of LE8 score and frailty using logistic regression models. The results were presented as odds ratios (*OR*s) with 95% confidence intervals (*CI*s). In models based on the quartiles of the LE8 score, we obtained *p*-values for linear trends by coding the quartiles as an ordinal variable (1, 2, 3, or 4). When the LE8 score was included in the models as a continuous variable, *OR*s for an absolute increment of 10 points in the LE8 score were computed. Three regression models were fitted to assess the associations between LE8 score and frailty. Model I was adjusted for age and sex. Model II was further adjusted for residency status, educational level, marital status, and drinking habits. Model III additionally adjusted for the history of hypertension, type 2 diabetes, heart disease, stroke, and the value of total cholesterol. Additionally, we employed 5-knot restricted cubic splines (RCS) to verify the assumption of linearity and explore the dose–response associations between the LE8 score and frailty through a likelihood ratio test.

We investigated the longitudinal associations between the LE8 score and incident frailty using Cox proportional hazards models for interval-censored data with the ICPHREG procedure. The same sets of covariates were included in the Cox proportional hazards models as in the cross-sectional analyses (Models I–III), to ensure consistency and comparability across analyses. Given the characteristics of routine health examination data, the time of frailty onset is likely to fall within the interval between two consecutive visits. Participants were treated as interval-censored between the health checkup dates confirming frailty and their last health checkup dates. Those who were not observed to have frailty at any health checkup were right-censored. To assess the robustness of the results, we conducted several sensitivity analyses that assumed the frailty incidence time was (i) the middle of the two health checkup intervals, (ii) the upper limit of the two intervals (considering the date of frailty as the date of the first health checkup showing frailty), and (iii) the lower limit of the two intervals, as suggested (24). Subgroup analyses were performed stratified by sex. A two-sided *p* < 0.05 was considered statistically significant. All analyses were conducted using SAS software (version 9.4), except for graphics, which were performed using the R package (version 4.4.1).

## Results

### Baseline characteristics of participants

A total of 59,654 older adults were included in the present study. Table 1 presents the baseline characteristics of participants according to sex-specific quartiles of the LE8 score. The mean (SD) baseline age was 70.1 (4.9) years, with 31,939 (53.5%) being women and 51,674 (86.6%) having non-local residency status. The mean (SD) LE8 score among participants was 74.5 (10.7). Compared to those in the lowest quartile, participants in the highest quartile of the LE8 score tended to be older, more likely to be female, local residents, have a higher education level, be married, not drink alcohol, and have a lower prevalence of hypertension, type 2 diabetes, and stroke. They also had lower total cholesterol levels and higher scores for tobacco exposure, physical activity, diet, sleep health, BMI, blood lipids, blood glucose, and blood pressure.

**Table 1:** Baseline characteristics of participants according to sex-specific quartiles of the LE8 score.

Characteristics	All participants (*n* = 59,654)	Quartiles of the LE8 score^a^				*P* value^b^
		First (*n* = 15,164)	Second (*n* = 15,209)	Third (*n* = 15,242)	Fourth (*n* = 14,039)	
Age, years, mean (SD)	70.1 (4.9)	69.9 (4.8)	70.0 (4.8)	70.2 (5.0)	70.1 (4.9)	<0.001
Sex						0.002
Male	27,715 (46.5)	7,069 (46.6)	6,999 (46.0)	7,262 (47.6)	6,385 (45.5)	
Female	31,939 (53.5)	8,095 (53.4)	8,210 (54.0)	7,980 (52.4)	7,654 (54.5)	
Residency status						<0.001
Local	7,980 (13.4)	1,776 (11.7)	2,015 (13.2)	2,166 (14.2)	2,023 (14.4)	
Non-local	51,674 (86.6)	13,388 (88.3)	13,194 (86.8)	13,076 (85.8)	12,016 (85.6)	
Educational level						<0.001
Less than high school	29,272 (49.1)	7,970 (52.6)	7,632 (50.2)	7,169 (47.0)	6,501 (46.3)	
High school or equivalent	27,263 (45.7)	6,603 (43.5)	6,841 (45.0)	7,195 (47.2)	6,624 (47.2)	
College and above	3,119 (5.2)	591 (3.9)	736 (4.8)	878 (5.8)	914 (6.5)	
Marital status						0.018
Married	57,983 (97.2)	14,689 (96.9)	14,781 (97.2)	14,832 (97.3)	13,681 (97.4)	
Other	1,671 (2.8)	475 (3.1)	428 (2.8)	410 (2.7)	358 (2.6)	
Drinking habits						<0.001
Never	47,663 (79.9)	11,373 (75.0)	11,968 (78.7)	12,327 (80.9)	11,995 (85.4)	
Seldom	7,414 (12.4)	2,257 (14.9)	1,991 (13.1)	1,845 (12.1)	1,321 (9.4)	
Daily	4,577 (7.7)	1,534 (10.1)	1,250 (8.2)	1,070 (7.0)	723 (5.1)	
Morbidity						
Hypertension	23,132 (38.8)	7,312 (48.2)	6,400 (42.1)	5,679 (37.3)	3,741 (26.6)	<0.001
Type 2 diabetes	9,672 (16.2)	3,730 (24.6)	2,785 (18.3)	2,269 (14.9)	888 (6.3)	<0.001
Heart disease	3,941 (6.6)	956 (6.3)	1,045 (6.9)	1,012 (6.6)	928 (6.6)	0.300
Stroke	2,966 (5.0)	824 (5.4)	760 (5.0)	777 (5.1)	605 (4.3)	<0.001
Total cholesterol, mmol/L, mean (SD)	5.2 (1.1)	5.6 (1.2)	5.3 (1.1)	5.0 (1.1)	4.7 (0.9)	<0.001
LE8 score, mean (SD)	74.5 (10.7)	60.6 (6.2)	71.8 (2.6)	78.9 (2.3)	87.9 (3.9)	<0.001
Tobacco exposure score	80.7 (35.8)	66.5 (44.1)	77.6 (37.5)	85.2 (30.9)	94.7 (18.2)	<0.001
Physical activity score	67.5 (43.7)	34.7 (44.1)	62.1 (44.7)	80.9 (35.3)	94.2 (19.9)	<0.001
Diet score	90.3 (29.6)	74.5 (43.6)	91.5 (27.9)	96.6 (18.2)	99.3 (8.6)	<0.001
Sleep health score	98.9 (10.3)	97.3 (16.3)	99.0 (10.1)	99.6 (6.5)	100.0 (2.1)	<0.001
BMI score	75.7 (23.0)	66.0 (23.4)	72.5 (22.8)	77.4 (21.9)	87.7 (18.0)	<0.001
Blood lipid score	64.7 (30.7)	50.2 (29.4)	60.2 (30.2)	68.0 (28.8)	81.8 (24.8)	<0.001
Blood glucose score	71.4 (27.0)	59.6 (26.9)	67.6 (26.3)	74.1 (25.6)	85.4 (22.0)	<0.001
Blood pressure score	47.1 (24.5)	35.8 (24.8)	43.9 (24.3)	49.7 (22.7)	60.0 (18.9)	<0.001

Values were shown in numbers (percentages) unless stated otherwise; SD, standard deviation; LE8, Life’s Essential 8; BMI, body mass index.

a Sex-specific quartiles of Life’s Essential 8 were 65.6, 73.8, and 81.3 in men and 68.8, 76.3, and 83.0 in women.

b Pearson’s chi-squared test or Kruskal–Wallis rank-sum test.

### Cross-sectional association between the LE8 score and frailty

**Figure 1:**
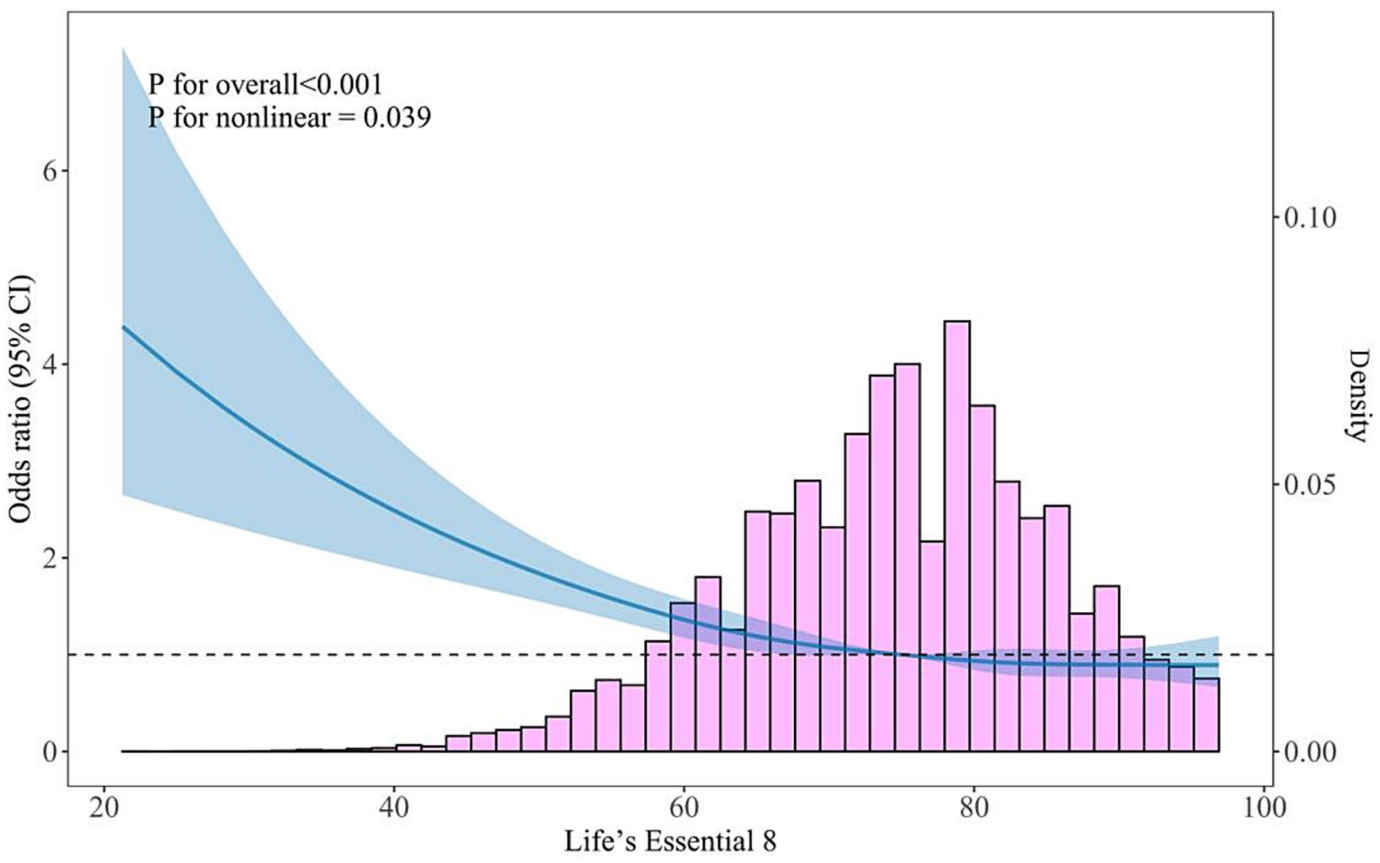
Restricted cubic spline plot examining the linearity assumption of the cross-sectional association between the LE8 score and frailty in the community-dwelling older adults. Odds ratio (solid blue lines) and 95% confidence intervals (shaded areas) were adjusted for age, sex, residency status, educational level, marital status, drinking habits, hypertension, type 2 diabetes, heart disease, stroke, and total cholesterol. Reference lines for no association are indicated by the dashed lines at an odds ratio of 1.0. The pink regions represent the histogram of the distribution of LE8 score. The model was conducted with 5 knots at the 5th, 27.5th, 50th, 72.5th, and 95th percentiles of Life’s Essential 8 (reference is the 50th percentile). CI, confidence interval.

According to the logistic regression models, compared with the first quartile of the LE8 score, the fully adjusted *ORs* of frailty were 0.77 (*95% CI*: 0.68, 0.87), 0.70 (*95% CI*: 0.61, 0.79), and 0.68 (*95% CI*: 0.58, 0.78) in the second, third, and fourth quartiles, respectively (Table 2). The logistic regression models showed significant linear trends in the quartiles of the LE8 score with frailty (*p* for trend < 0.001). Every 10-point increment in the LE8 score was associated with a 16% reduction in the odds of frailty (*OR*: 0.84, *95% CI*: 0.80, 0.88). These inverse associations persisted in subgroup analyses stratified by sex. Additionally, there was a significant inverse curvilinear trend for the association between LE8 score and frailty (*p* for nonlinear = 0.039, Figure 1).

**Table 2:** Cross-sectional associations between LE8 score and frailty assessed by logistic regression models.

Model	Quartiles of the LE8 score				*P* trend	Continuous^a^	*P* value
	First	Second	Third	Fourth			
Total							
No of cases/non-cases	616/14,548	475/14,734	433/14,809	356/13,683		1,880/57,774	
Model I	1.00	0.75 (0.66,0.85)	0.67 (0.59,0.76)	0.60 (0.52,0.68)	<0.001	0.81 (0.78,0.85)	<0.001
Model II	1.00	0.74 (0.66,0.84)	0.65 (0.57,0.74)	0.58 (0.51,0.67)	<0.001	0.81 (0.77,0.84)	<0.001
Model III	1.00	0.77 (0.68,0.87)	0.70 (0.61,0.79)	0.68 (0.58,0.78)	<0.001	0.84 (0.80,0.88)	<0.001
Men							
No of cases/non-cases	329/6,740	236/6,763	250/7,012	194/6,191		1,009/26,706	
Model I	1.00	0.69 (0.58,0.82)	0.69 (0.58,0.81)	0.60 (0.50,0.71)	<0.001	0.84 (0.79,0.89)	<0.001
Model II	1.00	0.67 (0.57,0.80)	0.67 (0.56,0.79)	0.57 (0.47,0.69)	<0.001	0.82 (0.78,0.87)	<0.001
Model III	1.00	0.69 (0.58,0.82)	0.72 (0.60,0.85)	0.66 (0.54,0.80)	<0.001	0.86 (0.81,0.92)	<0.001
Women							
No of cases/non-cases	287/7,808	239/7,971	183/7,797	162/7,492		871/31,068	
Model I	1.00	0.83 (0.70,0.99)	0.65 (0.54,0.79)	0.61 (0.51,0.75)	<0.001	0.79 (0.74,0.84)	<0.001
Model II	1.00	0.83 (0.69,0.98)	0.64 (0.53,0.77)	0.60 (0.50,0.74)	<0.001	0.79 (0.74,0.84)	<0.001
Model III	1.00	0.86 (0.72,1.03)	0.67 (0.55,0.82)	0.69 (0.56,0.86)	<0.001	0.81 (0.75,0.87)	<0.001

Model I: adjusted for age and sex; Model II: additionally adjusted for residency status, educational level, marital status, and drinking habits; Model III: additionally adjusted for the history of hypertension, type 2 diabetes, heart disease, stroke, and the value of total cholesterol. Values were shown in odds ratios (95% confidence intervals) unless stated otherwise; LE8, Life’s Essential 8.

a Odds ratio for an absolute increment of 10 points in the LE8 score.

### Longitudinal association between the LE8 score and frailty incidence

**Figure 2:**
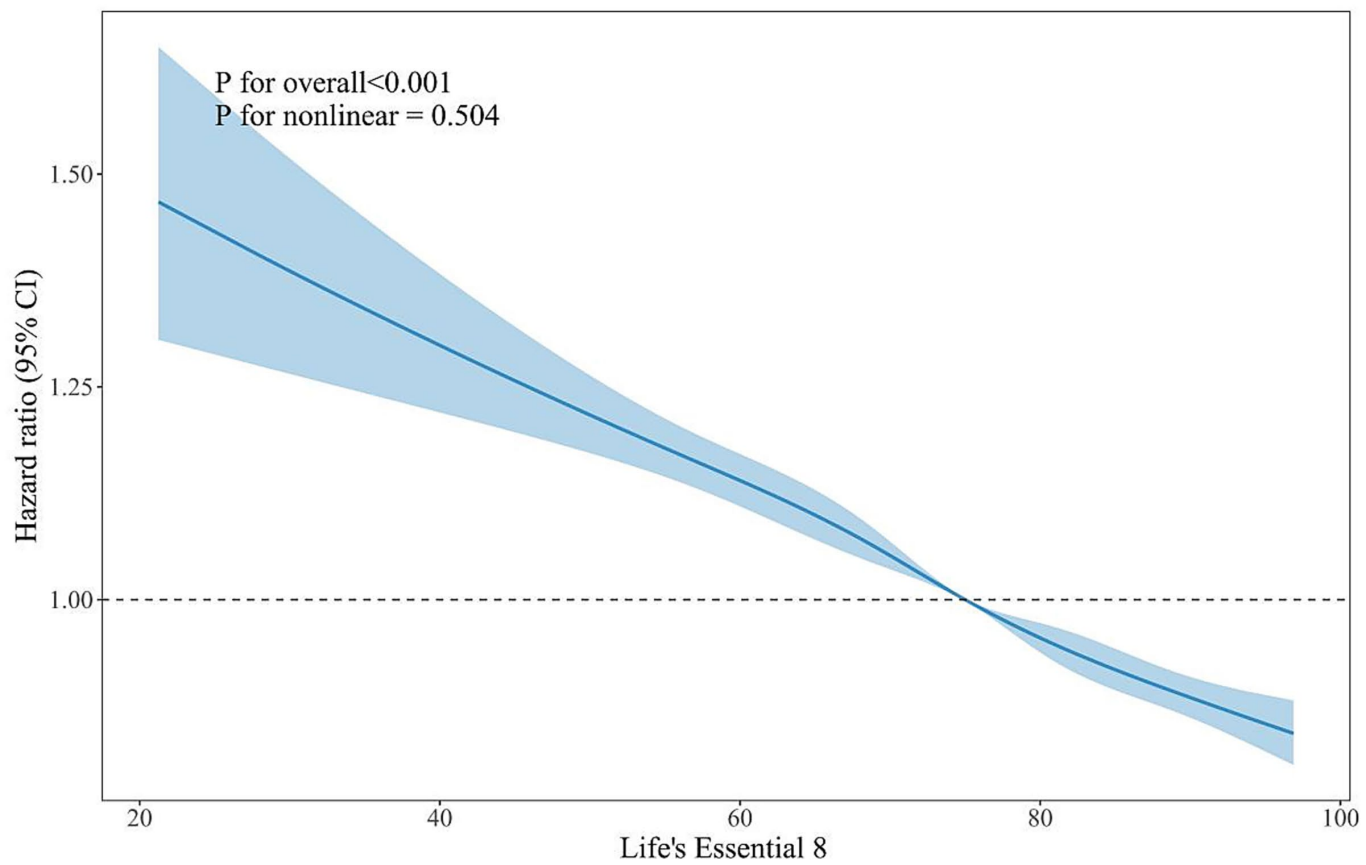
Restricted cubic spline plot examining the linearity assumption of longitudinal association between the LE8 score and frailty incidence in the community-dwelling older adults. Hazard ratio (solid lines) and 95% confidence levels (shaded areas) were adjusted for age, sex, residency status, educational level, marital status, drinking habits, hypertension, type 2 diabetes, heart disease, stroke, and total cholesterol. Reference lines for no association are indicated by the dashed lines at a hazard ratio of 1.00. The model was conducted with 5 knots at the 5th, 27.5th, 50th, 72.5th, and 95th percentiles of Life’s Essential 8 (reference is the 50th percentile). CI, confidence interval.

After a mean follow-up period of 1.21 years, 1,365 participants (2.4%) developed frailty. According to the Cox proportional hazards models, the LE8 score was significantly associated with frailty incidence after full adjustment for age, sex, residency status, educational level, marital status, drinking habits, hypertension, type 2 diabetes, heart disease, stroke, and total cholesterol (Table 3). The Cox proportional hazards models showed significant linear trends in quartiles of the LE8 score with frailty incidence (*p* for trend = 0.003). The linearity assumption between the LE8 score and the risk of frailty was confirmed by the RCS (*p* for nonlinear = 0.504, Figure 2). Every 10-point increment in the LE8 score was associated with an 11% reduction in the risk of frailty (*OR*: 0.89, *95% CI*: 0.84, 0.94). The results remained similar in the sensitivity analyses and subgroup analyses by sex (Supplementary Table S3).

**Table 3:** Longitudinal associations between the LE8 score and frailty incidence by Cox proportional hazards models.

Model	Quartiles of LE8 score				*P* trend	Continuous^a^	*P* value
	First	Second	Third	Fourth			
Total							
No of cases/non-cases	371/14,166	358/14,370	361/14,442	275/13,399		1,365/56,377	
Model I	1.00	0.88 (0.76,1.02)	0.83(0.71,0.96)	0.67 (0.57,0.78)	<0.001	0.86 (0.82,0.91)	<0.001
Model II	1.00	0.87 (0.75,1.00)	0.80 (0.70,0.93)	0.64 (0.55,0.75)	<0.001	0.85 (0.81,0.90)	<0.001
Model III	1.00	0.89 (0.78,1.03)	0.85 (0.74,0.99)	0.73 (0.62,0.87)	0.003	0.89 (0.84,0.94)	<0.001
Men							
No of cases/non-cases	184/6,552	183/6,578	203/6,805	156/6,031		726/25,966	
Model I	1.00	0.87 (0.71,1.06)	0.85 (0.70,1.04)	0.70 (0.56,0.86)	0.001	0.88 (0.83,0.94)	<0.001
Model II	1.00	0.85 (0.69,1.04)	0.83 (0.68,1.02)	0.67 (0.54,0.84)	<0.001	0.87 (0.82,0.93)	<0.001
Model III	1.00	0.86 (0.70,1.06)	0.89 (0.72,1.09)	0.76 (0.60,0.96)	0.038	0.91 (0.84,0.98)	0.012
Women							
No of cases/non-cases	187/7,614	175/7,792	158/7,637	119/7,368		639/30,411	
Model I	1.00	0.91 (0.74,1.12)	0.81 (0.66,1.01)	0.65 (0.52,0.82)	<0.001	0.85 (0.79,0.92)	<0.001
Model II	1.00	0.89 (0.73,1.10)	0.78 (0.63,0.97)	0.63 (0.50,0.79)	<0.001	0.84 (0.78,0.90)	<0.001
Model III	1.00	0.93 (0.75,1.14)	0.82 (0.66,1.03)	0.72 (0.56,0.93)	0.007	0.87 (0.80,0.95)	0.001

Model I: adjusted for age and sex; Model II: additionally adjusted for residency status, educational level, marital status, and drinking habits; Model III: additionally adjusted for the history of hypertension, type 2 diabetes, heart disease, stroke, and the value of total cholesterol. Values are hazard ratios (95% confidence intervals) unless stated otherwise; LE8, Life’s Essential 8.

a Hazard ratio for an absolute increment of 10 points in LE8 score.

## Discussion

In this large, extensive community-based study, we examined the cross-sectional and longitudinal associations between the LE8 score and frailty among older adults. Our findings revealed a significant inverse relationship between LE8 score and frailty in older women and men. Furthermore, a dose–response relationship was also identified between the LE8 score and frailty. We not only observed a significantly negative cross-sectional association between the LE8 score and frailty but also observed that a higher LE8 score was significantly associated with a lower risk of frailty. Our study will provide scientific evidence supporting the importance of CVH management in preventing frailty.

LE8, as a novel assessment tool for CVH status, has been demonstrated to be associated with reduced risks of diabetes mellitus in Chinese adults, as well as non-alcoholic fatty liver disease and hypertension in US adults (25–27). Previous studies reported that a 1-unit increment in the combined lifestyle factors (physical activity, dietary quality, and smoking) was associated with lower frailty (*β*: −0.62, *95% CI*: −0.84, −0.53) (28). Some metrics included in the LE8 have also been associated with a lower risk of frailty, such as healthy dietary habits, an appropriate BMI, recommended physical activity, and not smoking (29–32). In addition to lifestyle-related assessments, the LE8 also includes metabolic-related assessments, which provide a more comprehensive representation of overall CVH. A cross-sectional analysis involving 2,542 cancer survivors enrolled in the National Health and Nutrition Examination Surveys revealed that each unit increase in the LE8 score was associated with a 5% reduction in the odds of frailty (*OR*: 0.95, *95% CI*: 0.94, 0.96) (17). Similarly, our study observed this cross-sectional association and, for the first time, presents a dose–response association between the LE8 score and frailty in the community-dwelling older adults.

Interestingly, our RCS analysis in the cross-sectional data revealed a significant non-linear association between LE8 score and frailty. The curve suggested a steep decline in frailty risk as the LE8 score increased from lower to moderate levels, followed by a gradual flattening at higher LE8 scores. This finding implies a potential saturation effect, where initial improvements in CVH yield substantial benefits for frailty prevention, but additional gains beyond a certain level may provide diminishing returns. Such a pattern may reflect biological homeostasis in cardiovascular and metabolic systems, in which moderate enhancement in behaviors (e.g., physical activity, diet, and smoking cessation) and clinical factors (e.g., blood pressure and glucose) markedly reduce systemic inflammation and oxidative stress, whereas further optimization exerts relatively limited additional protection (33). In contrast, the longitudinal analysis showed a linear inverse relationship, indicating a consistent reduction in frailty incidence with increasing LE8 score during follow-up. Collectively, these findings imply that maintaining better CVH exerts protective effects against frailty, with the most pronounced benefit observed among individuals with poor or intermediate LE8 scores.

Our result aligns with previous cross-sectional evidence supporting an inverse curvilinear trend for the association between the LE8 score and depression, cognitive function, and metabolic syndrome, suggesting that lower CVH is associated with a higher adverse outcome compared to higher CVH (34–36). A prospective cohort study including 19,951 US adults aged 30–79 years identified approximately linear dose–response associations of increased total CVH metric score with reduced risk of all-cause and CVD-specific mortality (37). Our findings have important public health implications for China’s community-based healthcare system. Integrating the LE8 evaluation into these existing health checks could provide a simple, standardized, and actionable metric for monitoring CVH and identifying older adults at risk of frailty. This approach would allow community healthcare workers to deliver targeted lifestyle interventions—such as smoking cessation, dietary guidance, and physical activity promotion—within routine healthcare services. Furthermore, tracking the LE8 score over time could help evaluate the effectiveness of primary prevention programs and guide resource allocation for healthy aging initiatives.

The association between LE8 and frailty incidence is biologically plausible. LE8 incorporates factors such as unhealthy dietary habits, obesity, insufficient sleep, lipid abnormalities, and glucose metabolism disorders, all of which are associated with an increased release of pro-inflammatory factors (38–42). These pro-inflammatory markers include C-reactive protein, interleukin-6, and tumor necrosis factor-alpha, which have been observed in individuals with frailty and are known contributors to cardiovascular risk (16). These factors can reduce physiological reserve capacity and lead to dysregulation of various physiological systems, contributing to an imbalance that negatively affects overall health (43). In addition, inflammatory factors adversely affect CVH by promoting endothelial dysfunction and atherosclerosis while further reducing physiological reserve capacity and impairing stress response capabilities, contributing to the onset of frailty (44). By improving CVH through lifestyle modifications, LE8 may help mitigate the inflammatory processes that contribute to frailty.

An important consideration in examining the LE8 score and its association with frailty risk is the need to comprehensively account for lifestyle differences between sexes. Our baseline characteristics of participants align with findings from previous studies, which indicated that women tend to have higher LE8 scores than men (45, 46). Consequently, we analyzed the quartiles of the LE8 score separately by sex and adjusted for sex in subsequent models. Additionally, we stratified our analyses by sex and found consistent results across both groups. Collectively, these findings suggest that improving the LE8 score may reduce frailty risk irrespective of sex and serve as a useful screening tool to identify older adults at high risk for frailty, facilitating targeted frailty prevention and treatment strategies.

The strengths of this study included the large sample size and the longitudinal study design. We also fitted Cox proportional hazards models using the ICPHREG procedure in SAS, which specializes in analyzing interval-censored data. However, several limitations should be noted. First, since our study used self-reported information such as physical activity, smoking, and other variables to create the LE8 score and FI, classification errors or recall bias can occur when participants respond to the questionnaire. However, the self-report questionnaire used for assessing frailty has been validated (47). Second, due to limited data on more specific dietary factors in our study, we could not calculate the overall dietary quality score. Third, our participants were drawn from only one administrative district in Shenzhen, which could limit the generalizability of our findings. Nevertheless, it is well known that Shenzhen has the largest migrant population in China. Therefore, the results may be reliable and stable. Finally, some unmeasured factors, such as duration of residence and social support, may contribute to residual confounding.

## Conclusion

In this longitudinal study of community-dwelling older adults, we found that a higher LE8 score was consistently and significantly associated with both a lower prevalence of frailty and a reduced risk of incident frailty. These findings underscore the critical importance of maintaining optimal CVH for frailty prevention. The interventions focused on improving LE8 components (e.g., diet, physical activity, sleep, and blood pressure) may serve as an integrated and highly effective primary prevention strategy against frailty. Future research is needed to explore the causal relationship between LE8 score and frailty incidence.

## Data Availability

The original contributions presented in the study are included in the article/Supplementary material, further inquiries can be directed to the corresponding authors.
