# Correction: Cross-sectional and longitudinal associations between Life's Essential 8 and frailty in community-dwelling older adults

**DOI:** 10.3389/fpubh.2026.1792003

**Published:** 2026-01-22

**Authors:** Frontiers Production Office

**Affiliations:** Frontiers Media SA, Lausanne, Switzerland

**Keywords:** Life's Essential 8, frailty, older adults, community-dwelling, real-world data

Affiliation 2 “SUSTech Homeostatic Medicine Institute, School of Medicine, Southern University of Science and Technology, Shenzhen, China” was erroneously given as “School of Public Health and Emergency Management, Southern University of Science and Technology, Shenzhen, China”.

Affiliation 3 “Shenzhen Center for Disease Control and Prevention, Shenzhen, China” was erroneously given as “SUSTech Homeostatic Medicine Institute, School of Medicine, Southern University of Science and Technology, Shenzhen, China”.

Affiliation 4 “Shenzhen Maternity & Child Healthcare Hospital, Shenzhen, China” was erroneously given as “Shenzhen Center for Disease Control and Prevention, Shenzhen, China”.

The original version of this article has been updated.

