# Supplementary material for: Cross-sectional and longitudinal associations between Life’s Essential 8 and frailty in community-dwelling older adults
Source: Front Public Health. 2026 Jan 5;13:1730769. doi: 10.3389/fpubh.2025.1730769 (PMC12825033; doi:10.3389/fpubh.2025.1730769)
Supplement: Supplementary file 1 [file Table_1.docx]

**Supplementary Material:**


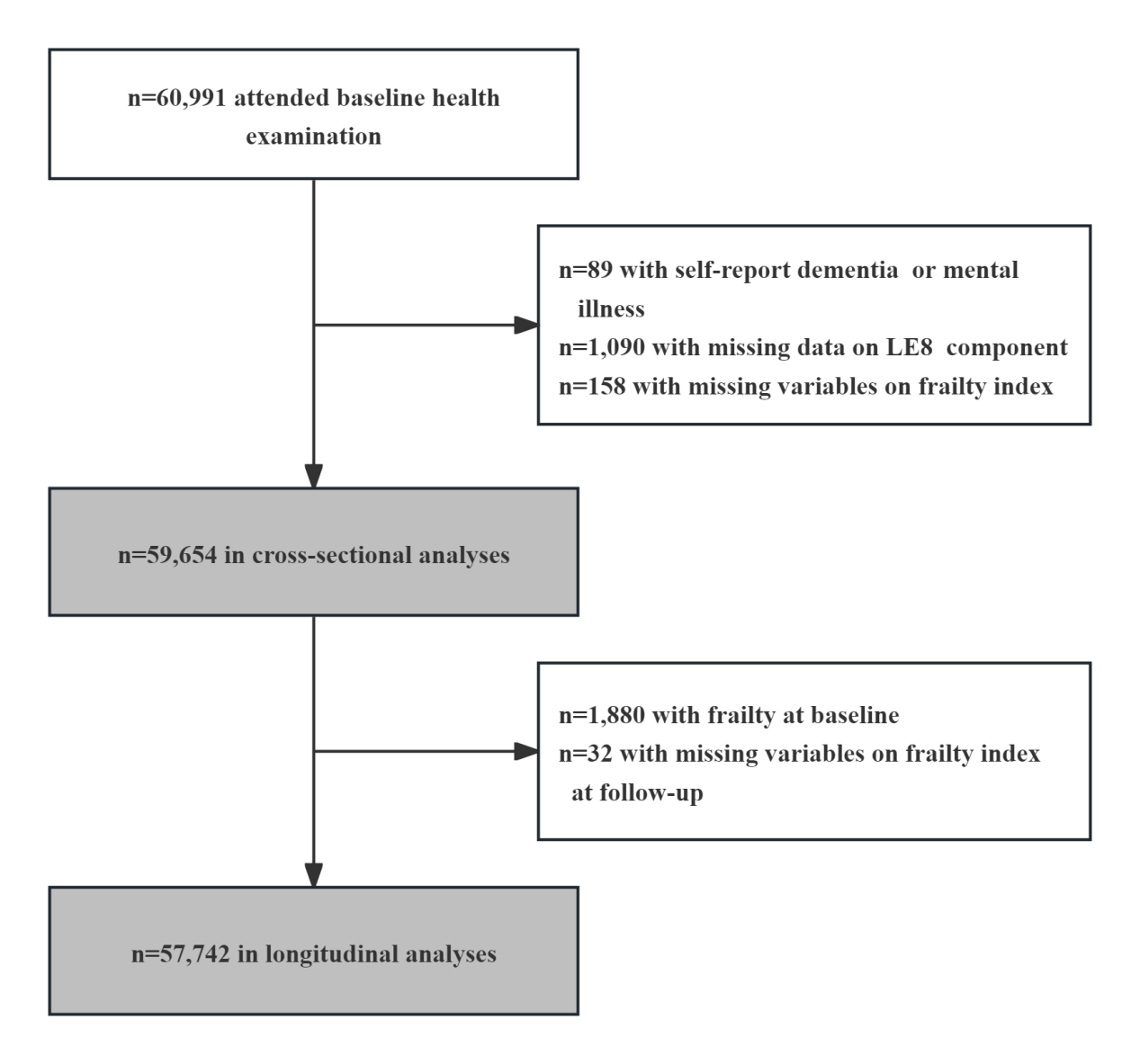
**Supplementary Figure S1. Flow-chart for participants used for the analyses**

**Supplementary Table S1. Definition of measurement and assessment of Life’s Essential 8 used in this study**

| Components | Method of measurement | Points of cardiovascular health metrics |
| --- | --- | --- |
| Nicotine exposure | Self-reported cigarettes smoking | 100: Never smoker  50: Former smoker  0: Current smoker |
| Physical activity | Self-reported physical activity habits | 100: Everyday  50: Seldom  0: Never |
| Diet | Self-reported diet habits | 100: Balanced  0: Meat-based or vegetable-based |
| Sleep health | Self-reported sleep problems | 100: No  0: Yes |
| Body mass index | Objective measurement of weight and height | 100: < 23.0  75: 23.0-24.9  50: 25.0-29.9  25: 30.0-34.9  0: ≥ 35.0 |
| Blood lipids | Plasma non–high-density lipoprotein cholesterol | 100: < 3.36  60: 3.36-4.13  40: 4.14-4.90  20: 4.91-5.68  0: ≥ 5.69 |
| Blood glucose | Self-reported diabetes and fasting blood glucose | 100: No history of diabetes with FBG < 5.6  60: No diabetes with FBG 5.6-6.9  40: Diabetes with FBG < 8.6  30: Diabetes with FBG 8.6-10.1  20: Diabetes with FBG 10.2-11.6  10: Diabetes with FBG 11.7-13.2  0: Diabetes with FBG ≥ 13.3 |
| Blood pressure | Objective measurement of systolic and diastolic blood pressure | 100: < 120 / < 80 (optimal)  75: 120-129 / < 80 (elevated)  50: 130-139 or 80-89 (stage 1 hypertension)  25: 140-159 or 90-99  0: ≥ 160 or ≥ 100 |

Note: Minor adaptations were applied to score nicotine exposure, physical activity, diet, and sleep health to match available variables in the NEPHSP dataset.

**Supplementary Table S2. Variables and cut-points for the construction of frailty index**

| **Variables** | **Coding** |
| --- | --- |
| **Diseases (n=11)** |  |
| Gallbladder disease | No=0, yes=1 |
| Kidney disease | No=0, yes=1 |
| Pulmonary diseases | No=0, yes=1 |
| Eye diseases | No=0, yes=1 |
| Thyroid disease | No=0, yes=1 |
| Fracture, osteoporosis | No=0, yes=1 |
| Arthritis | No=0, yes=1 |
| Anxiety and depression | No=0, yes=1 |
| Intervertebral disease | No=0, yes=1 |
| Hyperuricemia | No=0, yes=1 |
| Cancer | No=0, yes=1 |
| **Symptoms (n=7)** |  |
| Joint swelling and pain | No=0, yes=1 |
| Numbness of hands and feet | No=0, yes=1 |
| Pain | No=0, yes=1 |
| Constipation | No=0, yes=1 |
| Abdominal symptoms | No=0, yes=1 |
| Hearing loss | No=0, yes=1 |
| Visual impairment | No=0, yes=1 |
| **laboratory blood tests (n=9)** |  |
| Hemoglobin (g/L) | Woman: ≥110 =0, <110 =1; Man: ≥120 =0, <120 =1 |
| Platelet (1000 cells/uL) | Woman: 148-257 =0, else =1 ; Man:108-273 =0, else =1 |
| Alanine aminotransferase (U/L) | ≤40 =0, >40 =1 |
| Glutamic transaminase (U/L) | ≤40 =0, >40 =1 |
| Total bilirubin (μmol/L) | 3.4-17.1 =0, else =1 |
| Leukocyte (×10^9^/L) | 4-10 =0, else =1 |
| Serum creatinine (μmol/L) | ≤88.4 =0, >88.4 =1 |
| Blood urea | 2.9-8.2 =0, else =1 |
| Urinary protein | Negative =0, positive =1 |
| **Abdominal B-ultrasound** | Normal =0, abnormal =1 |
| **Self-rated health** | Very good=0, good=0.25, fair=0.50, poor=0.75, very poor=1 |
| **Activity of daily living** | Independent=0, mild dependent=0.33, Moderate dependent=0.67, dependent=1 |

**Supplementary Table S3. Sensitivity analyses for the longitudinal associations between the LE8 score and frailty incidence by Cox proportional hazard models**

| Model | Quartiles of LE8 score | | | | *P* trend | Continuous^a^ | *P* value |
| --- | --- | --- | --- | --- | --- | --- | --- |
|  | First | Second | Third | Fourth |  |  |  |
| Sensitivity analysis (i) |  |  |  |  |  |  |  |
| Total | 1.00 | 0.89 (0.77,1.03) | 0.85 (0.73,0.99) | 0.73 (0.62,0.87) | <.001 | 0.89 (0.84,0.94) | <.001 |
| Men | 1.00 | 0.86 (0.70,1.06) | 0.89 (0.72,1.09) | 0.76 (0.60,0.96) | 0.038 | 0.91 (0.84,0.98) | 0.012 |
| Women | 1.00 | 0.93 (0.76,1.15) | 0.82 (0.66,1.03) | 0.72 (0.55,0.92) | 0.007 | 0.87 (0.80,0.95) | 0.001 |
| Sensitivity analysis (ii) |  |  |  |  |  |  |  |
| Total | 1.00 | 0.87 (0.75,1.01) | 0.81 (0.70,0.95) | 0.69 (0.58,0.82) | <.001 | 0.87 (0.82,0.92) | <.001 |
| Men | 1.00 | 0.84 (0.68,1.03) | 0.84 (0.68,1.03) | 0.71 (0.56,0.90) | 0.007 | 0.89 (0.82,0.96) | 0.002 |
| Women | 1.00 | 0.92 (0.74,1.13) | 0.79 (0.63,0.99) | 0.68 (0.53,0.88) | 0.002 | 0.85 (0.78,0.93) | 0.001 |
| Sensitivity analysis (iii) |  |  |  |  |  |  |  |
| Total | 1.00 | 0.93 (0.80,1.08) | 0.92 (0.79,1.07) | 0.82 (0.69,0.97) | 0.031 | 0.93 (0.88,0.98) | 0.007 |
| Men | 1.00 | 0.92 (0.75,1.13) | 0.97 (0.79,1.20) | 0.87 (0.69,1.10) | 0.369 | 0.95 (0.89,1.03) | 0.213 |
| Women | 1.00 | 0.96 (0.78,1.18) | 0.87 (0.70,1.08) | 0.78 (0.60,1.00) | 0.040 | 0.90 (0.83,0.98) | 0.013 |

Sensitivity analyses defining the frailty incidence time is (i) the middle of the two health checkup intervals, (ii) the upper limit of two intervals, and (iii) the lower limit of two intervals; Models included adjustments for age, sex, residency status, educational level, marital status, drinking habits, hypertension, type-2 diabetes, heart disease, stroke, and total cholesterol; Values are hazard ratios (95% confidence intervals) unless stated otherwise; LE8: Life's Essential 8.

^a^Hazard ratio for an absolute increment of 10-point in LE8 score.
